# Proliferative glomerulonephritis with monoclonal immunoglobulin deposits: Successful treatment for new and rare entity

**DOI:** 10.1002/ccr3.3439

**Published:** 2020-11-11

**Authors:** Mouna Jerbi, Rym El Fatmi, Hanene Gaied, Dorra Belloumi, Lamia Torjemane, Raja Aoudia, Rim Goucha, Taieb Ben Abdallah, Tarek Ben Othman

**Affiliations:** ^1^ Department of Nephrology Mongi Slim Hospital La Marsa Tunisia; ^2^ Faculty of Medicine Tunis University of Tunis El Manar Tunis Tunisia; ^3^ Department of Hematology Centre National de Greffe de Moelle Osseuse Tunis Tunisia; ^4^ Department of Nephrology Charles Nicolle Hospital Tunis Tunisia

**Keywords:** Hematology, Nephrology, Pharmacology

## Abstract

Proliferative glomerulonephritis with monoclonal immunoglobulin deposits is a new disorder with undefined treatment modalities. We propose cyclophosphamide‐bortezomib‐dexamethasone and autologous stem cell transplantation as a therapeutic protocol.

## Graphical abstract

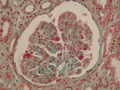

## 1 INTRODUCTION

Monoclonal gammopathy of renal significance (MGRS) is a heterogeneous entity of renal disorders defined in 2012.^1^ MGRS is related to the renal deposition and toxic effects of monoclonal immunoglobulin or its components (light and/or heavy chains), secreted from clonal B cells or plasma cells, that do not fit the criteria for a hematological malignancy. Monoclonal immunoglobulin may deposit into different parts of the kidney and leads to glomerular, tubulointerstitial, and vascular renal diseases.^2^, ^3^ Proliferative glomerulonephritis with monoclonal immunoglobulin deposits (PGNMID) is a relatively new and uncommon entity accounting of about 5.3% of MGRS.^3^, ^4^ In the field of PGNMID, monoclonal IgG deposits in glomeruli can produce a proliferative glomerulonephritis that mimics immune complex–mediated glomerulonephritis by light and electron microscopy. Proper recognition of this disease requires confirmation of monoclonality by immunofluorescence staining. There is no standard approach to manage these patients. We present here the first case of PGNMID successfully treated with bortezomib‐based induction followed by high‐dose melphalan and autologous stem cell transplantation (ASCT).

## 2 CASE REPORT

Our patient is a 43‐year‐old man with family history of laryngeal cancer in father and leukemia in mother, who consulted initially for headache and fatigue. Physical examination revealed high blood pressure. His doctor prescribed amlodipine. Eight months later, he was admitted to investigate anasarca and a history of macroscopic hematuria a week ago. He complained of a loss of appetite for 3 months. The physical examination revealed a blood pressure of 170/90 mm Hg, edema of lower limbs, and ascites. The urine was clear. Proteinuria 4+ and hematuria 3+ were detected on urinalysis. Initial workup showed anemia (Hb: 9.8 g/dL), normal platelet count, impure nephrotic syndrome (hypoproteinemia: 34 g/L, hypoalbuminemia: 13.5 g/L, and proteinuria: 25.4 g/d) due to hypertension, renal insufficiency (creatinemia: 154 µmol/L, MDRD: 45 mL/mn), and microscopic hematuria. Laboratories tests revealed glycemia: 4.57 mmol/L, calcemia: 2.49 mmol/L, hyperphosphatemia: 1.73 mmol/L, and hyperuricemia: 551 mmol/L (Table 1).

**Table 1:** Laboratory tests of the patient

Variable	Reference range	Patient value
Hb (g/dL)	13‐18	9.8
PLT (×10^3^/mm^3^)	150‐400	273
Sodium (mmol/L)	136‐146	136
Potassium (mmol/L)	3.5‐4.5	1.4
Chloride (mmol/L)	98‐107	103
Glucose (mmol/L)	3.88‐6.1	4.57
Calcium (mmol/l)	2.25‐2.6	2.49
Phosphorus (mmol/L)	0.74‐1.52	1.73
Uric Acid (mmol/L)	178‐416	551
Creatinine (µmol/L)	62‐105	154
Albumin (g/L)	35‐50	13.5
Total protein (g/lL)	60‐82	34
Aspartate aminotransferase (U/L)	5‐34	19
Alanine aminotransferase (U/L)	6‐55	11
Total bilirubin (mg/L)	3‐12	2.8
Alkaline phosphatase (U/L)	46‐116	49
Lactate dehydrogenase (U/L)	125‐220	204
C reactive protein (mg/L)	<8	2.7
Proteinuria (g/24 h)	< 0.3	25.4
Cryoglobulin		Negative
Hepatitis B serology		Negative
VIH serology		Negative
Hepatitis C serology		Negative
B2 microglobulin (ng/L)	1‐2.5	9.81
Complement C3 (g/L)	0.6‐1.8	1.15
CA‐125 (UI/mL)	<35	9
CA 19‐9 (UI/mL)	<37	16.7
Gamma globulin (g/L)	7.3‐13.9	4.8
Serum free light chain ratio		0.99

We then performed a renal biopsy that revealed a particular membranoproliferative glomerulonephritis (MPGN). There were 17 glomeruli all permeable, with some nodular glomerulosclerosis associated with a mesangial and endocapillary cell proliferation. The glomerular basement membranes were moderately thickened, split, or irregular. There were large aneurysmal dilatations and some intracapillary hyaline thrombi and capsular adhesion. Immunofluorescence demonstrated glomerular deposits that stained for a single light‐ and heavy‐chain isotype, IgGʎ (Figures 2, 3, 4, 5). There were also some segmental and mesangial IgM deposits and submembranous C3 deposits in 2 glomeruli (Figures 2, 3, 4, 5) In front of these histological results, etiological investigations were carried out. Cryoglobulinemia and hepatitis B serology were negative. Complement C3 was normal. Serum protein electrophoresis showed hypoglobulinemia: 4.8 g/L, and serum immuno‐electrophoresis revealed a monoclonal IgGʎ. Serum free light chain ratio was normal at 0.99 (K = 30.75 mg/L and lambda: 31.7 mg/L), and no urine free light chain was detected. Serum β2 microglobulin was 9.81mg/l (Table 1). Further evaluations did not confirm the diagnosis of multiple myeloma since the bone marrow evaluation revealed 4% plasma cells, the bone marrow biopsy was normal, albeit bone marrow flow cytometry was not performed, and there was no evidence of plasmacytoma. Bone X‐rays were normal. The spine magnetic resonance imaging was normal as well as the cardiac ultrasound. The patient had no extrarenal signs.

Hence, the patient was diagnosed as PGNMID subtype of MGRS.

**Figure 1:**
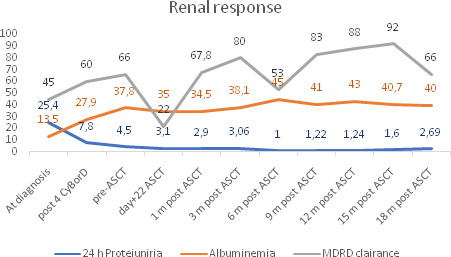
Renal response. CyBorD: cyclophosphamide‐bortezomib‐dexamethasone. ASCT: autologous stem cell transplantation

Treatment was initiated with four cycles of cyclophosphamide (orally, 500 mg/d; days 1, 8, and 15), bortezomib (subcutaneous, 1.3 mg/m^2^/day; days 1, 8, and 15), and dexamethasone (orally, 40 mg/day; days 1, 8, and 15). Hematological response was difficult to assess since baseline MIg was <10 g/L, and serum free light chain ratio and bone marrow examination were normal. Complete remission was defined as negative serum and urine immuno‐electrophoresis and normal free light chain ratio. Renal response was measured using the KDIGO practice guideline on glomerulonephritis. Complete renal response was defined as 0.5 g/day or less proteinuria, albuminemia > 30 g/L, and no more than 10% decrease in estimated glomerular filtration rate (eGFR) from baseline value. After 4 cycles of therapy, the patient obtained partial renal response with albuminemia at 27g/L, proteinuria at 7.85 g/d, and a creatinemia at 122mmol/L (MDRD clearance creatinine of 59.9 ml/mn) (Figure 1) Serum protein electrophoresis showed hypoproteinemia at 56g/L and a monoclonal spike at 9 g/L in the gamma‐globulin region. In front of this partial renal response and since the patient was in good general conditions, an ASCT was indicated. Peripheral blood stem cell mobilization protocol consisted of etoposide (375 mg/m^2^ days 1 and 2) and lenograstim (10 µg/kg/d from day 3 to day 11) leading to the collection of 19.3 10^6^ CD34+/Kg in 1 cytapheresis. Afterward, the patient received high‐dose melphalan (200mg/m2) followed by ASCT. Hematopoietic neutrophil reconstitution occurred on day 12 post‐ASCT. ASCT was well‐tolerated despite an acute renal failure at day 22 related to the administration of valacyclovir, as a prophylaxis for herpes simplex viruses. Post‐transplant evaluations revealed a stable normal creatinemia from 9 months post‐ASCT and a progressive reduction in proteinuria: 3.06 g/24H and 1.22 g/24H at 3 and 12 months post‐ASCT, respectively (Figure 1). The patient did not receive any maintenance therapy. At 30 months postdiagnosis and 18 months post‐transplant, the patient is alive with a status of partial renal response (proteinuria: 1.22 g/d, albuminemia: 40.7 g/L, clearance MDRD: 66 mL/mn). Consolidation with 2 cycles of cyclophosphamide‐bortezomib‐dexamethasone was currently indicated.

**Figure 2:**
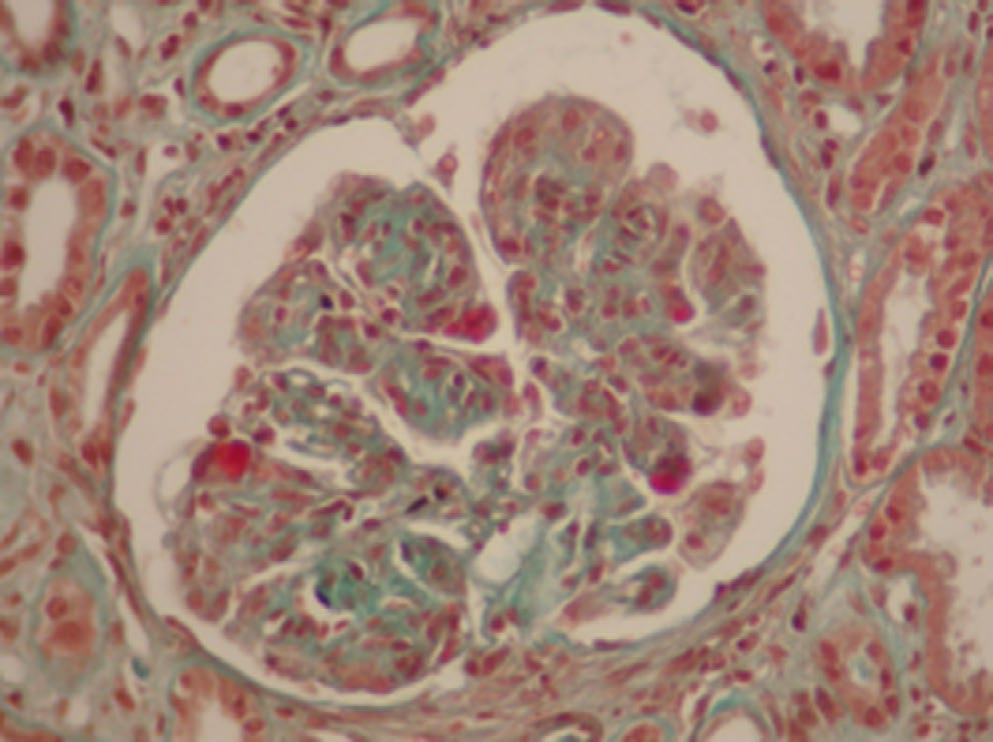
Renal biopsy, Masson's trichrome (magnification × 200). Nodular glomerulosclerosis and mesangial and endocapillary cell proliferation. Capsular adhesion

**Figure 3:**
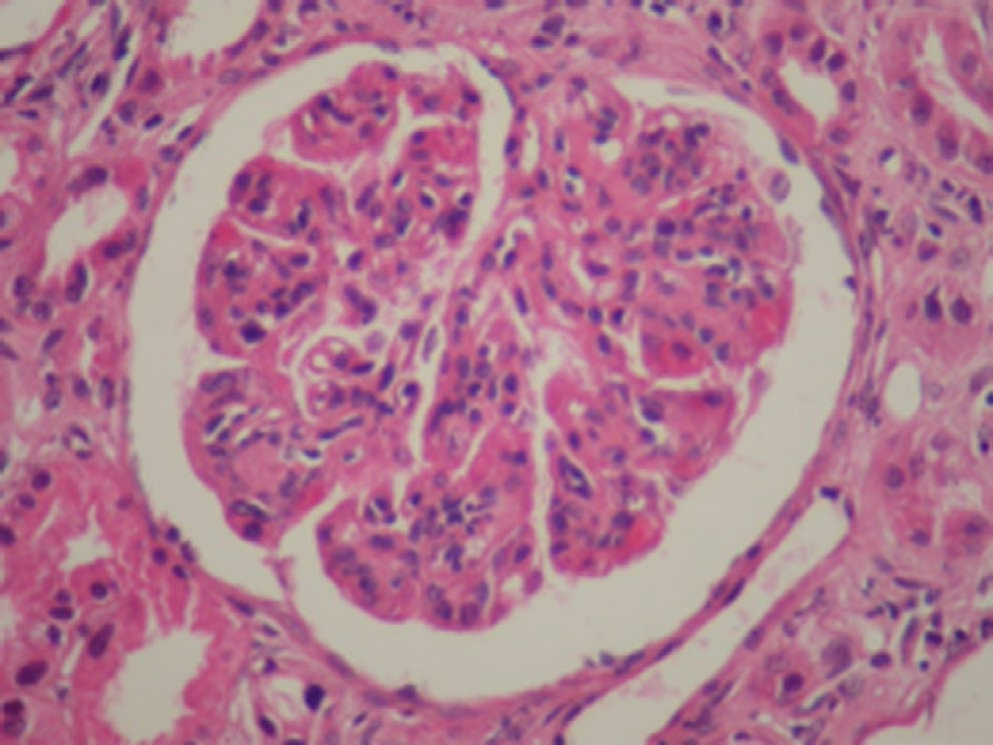
Renal biopsy, hematoxylin and eosin stain (magnification × 200). Endocapillary cell proliferation

**Figure 4:**
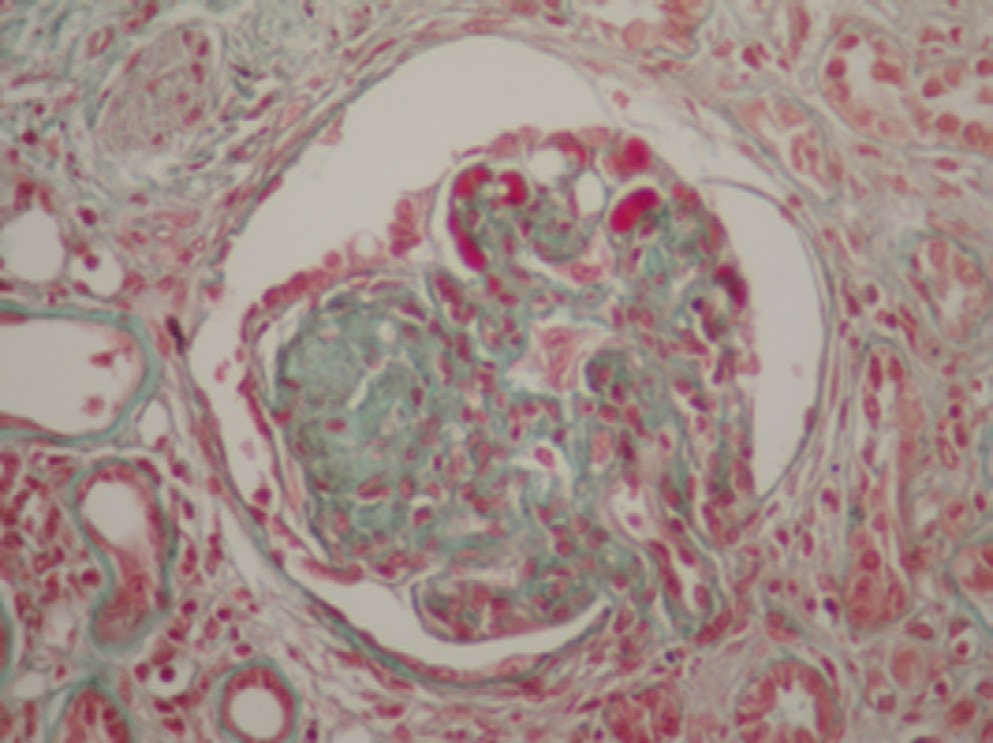
Renal biopsy, Masson's trichrome (magnification × 200). Nodular glomerulosclerosis. The podocytes that cover these segments present hypertrophy and hyperplasia

**Figure 5:**
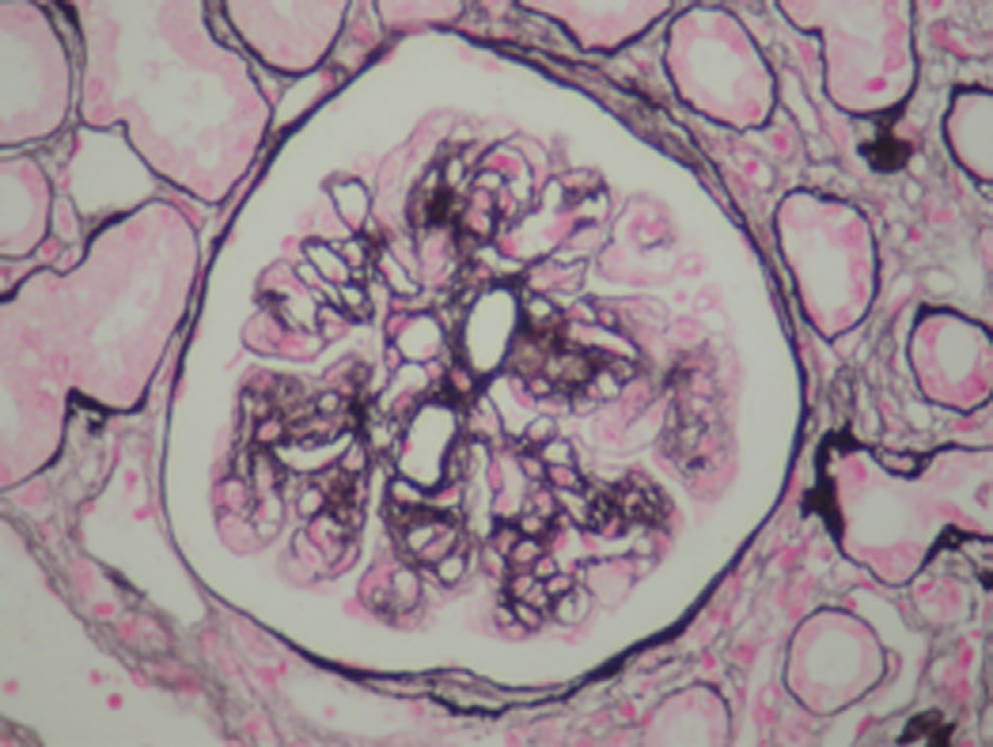
Renal biopsy, silver methenamine (Jones) (magnification × 200). The glomerular basement membranes are moderately thickened, split, or irregular. There are large aneurysmal dilations

## 3 DISCUSSION

We have presented the first case of PGNMID treated with bortezomib‐based induction followed by HDM/ASCT with obtaining a stable partial response. Although experts recommend cyclophosphamide‐bortezomib‐dexamethasone followed by high‐dose melphalan/ASCT in stage ≥ 3 CKD PGNMID, on review of the literature, no previous cases of PGNMID treated with this treatment modality were reported.

PGNMID represents a rare and newly diagnosed subtype of MGRS. PGNMID was first described by Nasr et al in 2004 when 10 patients were identified who had renal biopsy findings that showed unclassifiable proliferative or MPGN with monoclonal IgG deposits. On electron microscopy, mesangial, subendothelial, and/or subepithelial granular electron‐dense deposits were found.^5^ In larger and recent studies, biopsies most commonly showed diffuse proliferative or MPGN pattern (65%‐71%) on light microscopy. Immunofluorescence generally revealed single IgG subclass (56%‐62%) and a single light‐chain isotype (κ> λ) deposits restricted to the glomerulus. On electron microscopy, deposits were mostly subendothelial.^6^ Otherwise, histological pattern can be variable. Light microscopy may reveal membranous glomerulonephritis (25%) or mesangial nephritis (10%). IgM, IgA, and light‐chain deposits are observed in 21%, 13%, and 10%, respectively.^6^ Our patient had a particular form of PGNMID with a renal biopsy showing nodular glomerulosclerosis and monotypic IgG λ deposits only along the peripheral glomerular basement membranes and to a lesser extent in the nodules themselves.

In addition to a renal biopsy consistent with MPGN with IgG lambda–restricted deposits, our patient had the classical clinical presenting features of PGNMID, which included microscopic hematuria and proteinuria. Moreover, patients with PGNMID may also present nephrotic syndrome, end‐stage renal disease, and hypocomplementemia (mostly of the complement C3). In Javaugue's series, proteinuria, hematuria, and nephritic syndrome were noted in 100%, 78%, and 65% of cases, respectively. No patient had extrarenal signs as well as our patient, which represents a feature of the disease.^6^ Among the different types of renal diseases with monoclonal IgG deposition, PGNMID has one of the lowest rates of detection of both serum clonal dysproteinemia (25%‐50%) ^7^, ^8^ and the evidence of clonal disease in the bone marrow biopsies (25%).^9^ Our patient had evidence of dysproteinemia with a monoclonal serum IgG ʎ without evidence of clonal disease in the bone marrow.

The clinical course of PGNMID was reported by Nasr et al, who retrospectively assessed 37 PGNMID patients. They were treated with different regimens including prednisone alone, or associated with cyclophosphamide, thalidomide, or bortezomib. After a median follow‐up of 30 months, 38% had complete or partial renal recovery, 37% had persistent renal dysfunction, and 22% progressed to end‐stage renal disease.^9^ Moreover, reported cases of PGNMID treated with various therapies are published.^10^, ^11^ Details regarding different initial treatment regimens and responses are presented in Table 2.

**Table 2:** Proliferative glomerulonephritis with monoclonal immunoglobulin deposits: initial treatment regimens and responses

Ref.	Patient sex/age	eGFR	Proteinuria (g/day)	Renal biopsy deposits	Circulating paraprotein	Detectable clone on bone marrow biopsy	Treatment	Evolution
Noto ^10^	M/75	‐	6	IgG kappa	IgG kappa	Yes	4 mo BOR/D followed by BOR maintenance	CR after 4 mo + histological response after 8 mo
Gumpber ^11^ pt 4	M/53	45	3	IgG kappa	IgG kappa	Yes	6 mo CY/BOR/D	PR after 5.1 mo CR after 33.3 mo Relapse after 18 mo of 1st CR retreated with BOR/D with CR
Gumpber ^11^ pt 15	M/69	38	3.4	IgG lambda	IgG lambda	No	6mo RTX/CY/BOR/D	PR after 1.1 mo CR after 6.5 mo
Gumpber ^11^ pt 11	M/34	51	15	IgG lambda	No	No	6 mo RTX/PRED	PR after 9 mo Relapse at 12 mo
Gumpber ^11^ pt 16	F/75	14	1.47	IgG kappa	No	No	6 mo BOR/D	PR after 3 mo

Abbreviations: BOR, bortezomib; CR, complete response; CY, cyclophosphamide; D, dexamethasone; eGFR, estimated glomerular filtration rate; F, female; M, male; mo, month; PR, partial response; pt, patient; PRED, prednisone; RTX, rituximab.

Since randomized controlled studies or prospective studies are not yet available in the field of MGRS, treatment recommendations are mainly based on expert consensus opinion and clinical experiences with small numbers of patients.^12^, ^13^ As MGRS may be driven by clonal plasma cells, although not always detectable by current techniques, classical antimyeloma therapy is recommended. High‐dose melphalan/ASCT for eligible patients under the age of 65 years has the potential to offer deep and durable remissions since the plasma cell burden is typically low. In the case of PGNMID, experts’ recommendations on initiation of therapy are based on the stage of chronic kidney disease and the degree of proteinuria and high‐dose melphalan/ASCT is indicated in eligible patients with stage ≥ 3 chronic kidney disease.^12^ At diagnosis, our patient had stage 3 chronic kidney disease and 25.4 g of proteinuria per day, meeting indications for starting treatment with four cycles of cyclophosphamide‐bortezomib‐dexamethasone followed by high‐dose melphalan/ASCT. Before transplantation, he had stage 4 chronic kidney disease and melphalan was used at the standard dose of 200mg/m^2^ without any unexpected toxicities despite an acute renal failure at day 22 related to the administration of valacyclovir. Consolidation by high‐dose melphalan and ASCT should be considered in eligible PGNMID patients since relapse or progression of the disease may occur rapidly after a cyclophosphamide‐bortezomib‐dexamethasone induction and even on bortezomib maintenance.^14^ Although melphalan/ASCT is feasible in patient with renal failure, mortality and morbidity including the risk of worsening renal function increase with the severity of renal impairment. Thus, melphalan dose should be adjusted in patients with chronic kidney disease stage 3 or above and risk/benefit ratio should be carefully evaluated in each case.^12^ At 18 months after treatment (30 months after diagnosis), our patient presented persistent monoclonal immunoglobulin detected by serum immuno‐electrophoresis and proteinuria: 2.69 g/day (<3.5 g/d). Thus, he was in very good partial hematological response and partial renal response. The evaluation of hematological response is crucial in MGRS since it impacts the renal response and survival (Chauvet et al). In PGNMID and others MGRS due to the deposition of intact monoclonal immunoglobulin, it is recommended to use multiple myeloma criteria to evaluate the hematological response. These criteria are valid if causal monoclonal immunoglobulin and/or bone marrow clonal disease is detectable. Renal response is usually delayed and should be regularly monitored using the KDIGO practice guideline.^15^

## 4 CONCLUSION

We reported the case of a patient presenting proliferative glomerulonephritis with monoclonal immunoglobulin deposits, an uncommon monoclonal gammopathy of renal significance. This case stressed the importance of a rapid diagnosis and classification of these disorders by renal biopsy and showed the feasibility and efficacy of cyclophosphamide‐bortezomib‐dexamethasone treatment followed by high‐dose melphalan and autologous stem cell transplantation in proliferative glomerulonephritis with monoclonal immunoglobulin deposits.

## CONFLICT OF INTEREST

None declared.

## AUTHOR CONTRIBUTIONS

Mouna Jerbi and Rym El Fatmi: collected data and wrote the first draft of the article. Hanene Gaied, Dorra Belloumi, and Lamia Torjemene: critically reviewed and edited drafts. Taieb Ben Abdallah and Tarek Ben Othman: made the last corrections and approved the final manuscript. Raja Aoudia and Rim Goucha: were the pathologists and reviewed drafts.

## ETHICAL APPROVAL

The patient has given his written informed consent to publish his case including publication of images.

## References

[1] Leung N , Bridoux F , Hutchison CA , et al. Monoclonal gammopathy of renal significance: when MGUS is no longer undetermined or insignificant. Blood. 2012;120:4292‐4295. 23047823 10.1182/blood-2012-07-445304

[2] Correia SO , Santos S , Malheiro J , Cabrita A , Martins LS , Santos J . Monoclonal gammopathy of renal significance: Diagnostic workup. World J Nephrol. 2017;6(2):72‐78. 28316940 10.5527/wjn.v6.i2.72 PMC5339639

[3] Xiao‐juanYu XZ , Li D‐Y . Renal pathologic spectrum and clinical outcome of monoclonal gammopathy of renal significance: a large retrospective case series study from a single institute in China. Nephrology. 2020;25(3):202‐211. 31301197 10.1111/nep.13633

[4] Khera A , Panitsas F , Djebbari F , Kimberger K , Stern S , Quinn J . Long term outcomes in monoclonal gammopathy of renal significance. Br J Haematol. 2019;186(5):706‐716. 31141168 10.1111/bjh.15987

[5] Nasr SH , Markowitz GS , Stokes MB , et al. Proliferative glomerulonephritis with monoclonal IgG deposits: A distinct entity mimicking immune‐complex glomerulonephritis. Kidney Int. 2004;65:85‐96. 14675039 10.1111/j.1523-1755.2004.00365.x

[6] Javaugue V , Bouteaua I , Sirac C , et al. Classification et prise en charge thérapeutique des gammapathies monoclonales de signification rénale. Rev Med Interne. 2017;39(3):161–170. 28457684 10.1016/j.revmed.2017.03.012

[7] Geldenhuys L , Jones B . Proliferative Glomerulonephritis with monoclonal IgG deposits. J Am Soc Nephrol. 2009;20(9):2055‐2064. 19470674 10.1681/ASN.2009010110 PMC2736767

[8] Bhutani G , Nasr SH , Said SM , et al. Hematologic characteristics of proliferative glomerulonephritis with nonorganized monoclonal immunoglobulin deposits. Mayo Clin Proc. 2015;90:587‐596. 25939936 10.1016/j.mayocp.2015.01.024

[9] Nasr SH , Satoskar A , Markowitz GS , et al. Proliferative glomerulonephritis with monoclonal IgG deposits. J Am Soc Nephrol. 2009;20:2055‐2064. 19470674 10.1681/ASN.2009010110 PMC2736767

[10] Noto R , Kamiura N , Ono Y , et al. Successful treatment with bortezomib and dexamethasone for proliferative glomerulonephritis with monoclonal IgG deposits in multiple myeloma: a case report. BMC Nephrol. 2017;18(127):1‐6. 28385149 10.1186/s12882-017-0524-7 PMC5382661

[11] Gumber R , Cohen JB , Palmer MB . A clone‐directed approach may improve diagnosis and treatment of proliferative glomerulonephritis with monoclonal immunoglobulin deposits. Kidney Int. 2018;94(1):199–205. 29759418 10.1016/j.kint.2018.02.020

[12] Fermand JP , Bridoux F , Kyle RA , et al. How I treat monoclonal gammopathy of renal significance (MGRS). Blood. 2013;122:3583‐3590. 24108460 10.1182/blood-2013-05-495929

[13] Caravaca‐Fontána F , Gutiérreza E , Delgado Lillob R , Pragaa M . Monoclonal gammopathies of renal significance. Nefrologia. 2017;37(5):465‐477. 28946960 10.1016/j.nefro.2017.03.012

[14] Lee H , Duggan P , Neri P , Tay J , Jimenez‐Zepeda VH . Bortezomib Maintenance for the Treatment of Monoclonal Gammopathy of Renal Significance. Mediterr J Hematol Infect Dis. 2019;11(1):e2019007. 30671213 10.4084/MJHID.2019.007 PMC6328037

[15] Radhakrishnan J , Cattran DC . The KDIGO practice guideline on glomerulonephritis: reading between the (guide)lines–application to the individual patient. Kidney Int. 2012;82(8):840‐856. 22895519 10.1038/ki.2012.280

